# Supplementary material for: Bayesian refinement of protein structures and ensembles against SAXS data using molecular dynamics
Source: PLoS Comput Biol. 2017 Oct 18;13(10):e1005800. doi: 10.1371/journal.pcbi.1005800 (PMC5662244; doi:10.1371/journal.pcbi.1005800)
Supplement: S1 Archive — (BZ2) [file pcbi.1005800.s011.tar.bz2 › gromacs_4.62_bayesian_saxs_refi/share/html/online/g_dipoles.html]

g\_dipoles


| g\_dipoles Main Table of Contents |

**VERSION 4.6  
Sat 19 Jan 2013**

---

### Description

g\_dipoles computes the total dipole plus fluctuations of a simulation
system. From this you can compute e.g. the dielectric constant for
low-dielectric media.
For molecules with a net charge, the net charge is subtracted at
center of mass of the molecule.

The file Mtot.xvg contains the total dipole moment of a frame, the
components as well as the norm of the vector.
The file aver.xvg contains <|μ|^2> and |<μ>|^2 during the
simulation.
The file dipdist.xvg contains the distribution of dipole moments during
the simulation
The value of -mumax is used as the highest value in the distribution graph.

Furthermore, the dipole autocorrelation function will be computed when
option -corr is used. The output file name is given with the -c
option.
The correlation functions can be averaged over all molecules
(mol), plotted per molecule separately (molsep)
or it can be computed over the total dipole moment of the simulation box
(total).

Option -g produces a plot of the distance dependent Kirkwood
G-factor, as well as the average cosine of the angle between the dipoles
as a function of the distance. The plot also includes gOO and hOO
according to Nymand & Linse, J. Chem. Phys. 112 (2000) pp 6386-6395. In the same plot,
we also include the energy per scale computed by taking the inner product of
the dipoles divided by the distance to the third power.

EXAMPLES

g\_dipoles -corr mol -P 1 -o dip\_sqr -mu 2.273 -mumax 5.0

This will calculate the autocorrelation function of the molecular
dipoles using a first order Legendre polynomial of the angle of the
dipole vector and itself a time t later. For this calculation 1001
frames will be used. Further, the dielectric constant will be calculated
using an -epsilonRF of infinity (default), temperature of 300 K (default) and
an average dipole moment of the molecule of 2.273 (SPC). For the
distribution function a maximum of 5.0 will be used.

### Files

| option | filename | type | description |
| --- | --- | --- | --- |
| **-en** | ener.edr | Input, Opt. | Energy file |
| **-f** | traj.xtc | Input | Trajectory: xtc trr trj gro g96 pdb cpt |
| **-s** | topol.tpr | Input | Run input file: tpr tpb tpa |
| **-n** | index.ndx | Input, Opt. | Index file |
| **-o** | Mtot.xvg | Output | xvgr/xmgr file |
| **-eps** | epsilon.xvg | Output | xvgr/xmgr file |
| **-a** | aver.xvg | Output | xvgr/xmgr file |
| **-d** | dipdist.xvg | Output | xvgr/xmgr file |
| **-c** | dipcorr.xvg | Output, Opt. | xvgr/xmgr file |
| **-g** | gkr.xvg | Output, Opt. | xvgr/xmgr file |
| **-adip** | adip.xvg | Output, Opt. | xvgr/xmgr file |
| **-dip3d** | dip3d.xvg | Output, Opt. | xvgr/xmgr file |
| **-cos** | cosaver.xvg | Output, Opt. | xvgr/xmgr file |
| **-cmap** | cmap.xpm | Output, Opt. | X PixMap compatible matrix file |
| **-q** | quadrupole.xvg | Output, Opt. | xvgr/xmgr file |
| **-slab** | slab.xvg | Output, Opt. | xvgr/xmgr file |

### Other options

| option | type | default | description |
| --- | --- | --- | --- |
| **-[no]h** | bool | no | Print help info and quit || **-[no]version** | bool | no | Print version info and quit || **-nice** | int | 19 | Set the nicelevel || **-b** | time | 0 | First frame (ps) to read from trajectory || **-e** | time | 0 | Last frame (ps) to read from trajectory || **-dt** | time | 0 | Only use frame when t MOD dt = first time (ps) || **-[no]w** | bool | no | View output .xvg, .xpm, .eps and .pdb files || **-xvg** | enum | xmgrace | xvg plot formatting: xmgrace, xmgr or none || **-mu** | real | -1 | dipole of a single molecule (in Debye) || **-mumax** | real | 5 | max dipole in Debye (for histogram) || **-epsilonRF** | real | 0 | ε of the reaction field used during the simulation, needed for dielectric constant calculation. WARNING: 0.0 means infinity (default) || **-skip** | int | 0 | Skip steps in the output (but not in the computations) || **-temp** | real | 300 | Average temperature of the simulation (needed for dielectric constant calculation) || **-corr** | enum | none | Correlation function to calculate: none, mol, molsep or total || **-[no]pairs** | bool | yes | Calculate \|cos(θ)\| between all pairs of molecules. May be slow || **-ncos** | int | 1 | Must be 1 or 2. Determines whether the  is computed between all molecules in one group, or between molecules in two different groups. This turns on the -g flag. || **-axis** | string | Z | Take the normal on the computational box in direction X, Y or Z. || **-sl** | int | 10 | Divide the box into this number of slices. || **-gkratom** | int | 0 | Use the n-th atom of a molecule (starting from 1) to calculate the distance between molecules rather than the center of charge (when 0) in the calculation of distance dependent Kirkwood factors || **-gkratom2** | int | 0 | Same as previous option in case ncos = 2, i.e. dipole interaction between two groups of molecules || **-rcmax** | real | 0 | Maximum distance to use in the dipole orientation distribution (with ncos == 2). If zero, a criterion based on the box length will be used. || **-[no]phi** | bool | no | Plot the 'torsion angle' defined as the rotation of the two dipole vectors around the distance vector between the two molecules in the .xpm file from the -cmap option. By default the cosine of the angle between the dipoles is plotted. || **-nlevels** | int | 20 | Number of colors in the cmap output || **-ndegrees** | int | 90 | Number of divisions on the y-axis in the cmap output (for 180 degrees) || **-acflen** | int | -1 | Length of the ACF, default is half the number of frames || **-[no]normalize** | bool | yes | Normalize ACF || **-P** | enum | 0 | Order of Legendre polynomial for ACF (0 indicates none): 0, 1, 2 or 3 || **-fitfn** | enum | none | Fit function: none, exp, aexp, exp\_exp, vac, exp5, exp7, exp9 or erffit || **-ncskip** | int | 0 | Skip this many points in the output file of correlation functions || **-beginfit** | real | 0 | Time where to begin the exponential fit of the correlation function || **-endfit** | real | -1 | Time where to end the exponential fit of the correlation function, -1 is until the end |

---

http://www.gromacs.org  
gromacs@gromacs.org
